# AI and digital technology paradigm for seniors: a Singapore lens to healthy longevity

**DOI:** 10.3389/fpubh.2024.1487139

**Published:** 2025-01-15

**Authors:** Chuan De Foo, Logan Krishaa, Beatrix Tang Si Ik, Ewa Kosycarz, Jinyuan Wang, Ken Wah Teo

**Affiliations:** ^1^Saw Swee Hock School of Public Health, National University of Singapore and National University Health System, Singapore, Singapore; ^2^Duke-NUS Medical School, Singapore, Singapore; ^3^Warsaw School of Economics, Warsaw, Poland; ^4^National University of Singapore, Singapore, Singapore; ^5^MOH Office for Healthcare Transformation, Singapore, Singapore; ^6^Preventive Medicine, National University Health System, Singapore, Singapore; ^7^Division of Population Health and Integrated Care, Singapore General Hospital, Singapore, Singapore

**Keywords:** digital health, artificial intelligence (AI), health system, Singapore, aging population

## The shifting sands of health

Living longer and free of morbidity should not be an esoteric ideology, owing to advances in public health and medical science. However, societies as a whole remain dismally unprepared for this demographic transition. The Global Roadmap for Healthy Longevity calls for a multidisciplinary effort to shift the focus from merely coping with aging populations to enabling successful and sustainable aging for all (1, 2). Artificial intelligence and other digital technologies (AIDTs) stand out as pivotal forces with the potential to drive a paradigm shift in healthcare by improving medical care, addressing social isolation, enhancing mental wellness for seniors and augmenting the healthcare workforce.

The toll of aging cannot be dismissed. Pervasive public health concerns, such as loneliness and social exclusion experienced by seniors, continue to contribute to a myriad of health impairments (3–5). Ultimately, neglecting non-medical determinants of health directly exacerbates age-related illnesses, depriving seniors of healthy, fulfilling years of life (3, 4). In this digital era, where social interactions are increasingly taking place in the digital space, older adults often find themselves excluded, albeit unintentionally, due to a lack of tech-savviness and access to digital technology (6).

## Bridging the inevitable technological divide: evolving strategies

Seniors are often stereotyped as being less receptive to new technologies. However, having been born into an analog world, they face a stark contrast to the digital landscape in which younger generations have grown up. The rise of digitalisation in healthcare has taken an emotional toll on seniors, leading to feelings of inadequacy, isolation and diminished self-efficacy. Limited digital literacy, physical challenges and unfamiliarity impede their ability to effectively utilize the wave of health applications such as telemedicine, health monitoring wearables and online payment claimant systems, restricting their autonomy in managing their health (7). As governments recognize the indisputable economic and healthcare burden attributed to this group, more health and non-health initiatives aimed at digitally aiding older adult health have been implemented. Singapore stands out in this regard, particularly in its use of AI.

The Silver Infocomm Initiative in Singapore exemplifies this by hosting intergenerational IT boot camps, where tech-savvy young adults are paired with older adults, providing a comfortable environment for acquiring digital skills, engaging with health and social online applications and gaining self-efficacy in the digital climate (8). Such initiatives are also widely implemented in neighborhood Active Aging Centers and CareCorner, both serving as one-stop shops for seniors to receive paraclinical services and social empowerment training (9). Linking seniors to such facilities that are easily reachable due to their proximity to residential estates promotes access to senior-friendly digital literacy activities. Furthermore, such interactions foster intergenerational connectivity and shift ageist perspectives by facilitating the mutual exchange of knowledge, expertise, values, and skills. Another facet of the campaign promotes a culture of volunteerism through the training of seniors as Silver Infocomm Wellness Ambassadors (SIWAs) (10, 11). SIWAs inspire and encourage their peers to embrace digital technology for social networking, blogging, discovering online communities with shared interests and using platforms for critical tasks such as e-banking and telemedicine. SIWAs create a more empathetic space, especially for more reserved older adults, empowering them to stay connected and informed in today's digital age.

It is important to recapitulate that technology is not a substitute for human connection, but it can greatly assist in establishing and maintaining a relationship. Intergenerational bonding, such as spending time with one's grandchildren and video gaming together, significantly enhances the biopsychosocial wellbeing of older adults by creating shared experiences, developing mutual compassion and empathy and keeping them cognitively stimulated and socially connected (12). Another good example is Kinsome, an AI application that facilitates online interactions between grandparents and their grandchildren by offering customized icebreakers, shared activities and in-app interactions designed to encourage bonding and strengthen relationships (13). A separate application, MaestroAI, utilizes the PoseNet machine learning model to digitalise and gamify tai chi, bringing together people of different ages to tackle depression and the lack of intergenerational interaction (14).

Delving into mental health innovations, Lions Befrienders, a social service agency, utilizes AI through the Opsis Emotion AI service to analyse the facial expressions of seniors during video calls, detecting non-verbal cues of stress, depression and anxiety to provide targeted mental health services (15, 16). During the COVID-19 pandemic, Lions Befrienders also distributed devices to older adults, enabling communication to prevent social isolation, providing medication reminders and facilitating teleconsultations (17). This enhanced accessibility to digital health and increased familiarity empowers older Singaporeans to take charge of their health by providing avenues to meet their biopsychosocial needs. Nonetheless, despite such targeted initiatives, broader challenges remain. A survey by the Singapore Eye Research Institute (SERI) found that while telemedicine could reduce unnecessary clinic visits, 55% of seniors over 60 were unlikely to adopt digital health services, primarily due to technological difficulties and a lack of trust in AI (18). Overcoming this requires a multi-faceted approach, including live demonstrations, caregiver support and user-friendly interfaces to make digital inclusion a standard for equitable healthcare access.

## AIDT policies must not be tokenistic

Despite the benefits AIDTs bring, the health system must be aware of their unintended consequences. Firstly, there is some level of irony in the digital marvel of AI and IT; in reality, they have been shown to result in more social isolation. From early models such as ELIZA and PARRY in the 1960s and 1970s to not-so-modern advancements such as EBER and Charlie, there have been vast improvements in the ability of chatbots to provide empathetic companionship and cognitive support to seniors (19). However, all that glitters is not gold—the allure of digital alternatives poses significant risks. Some, powerful enough to mimic the voices, faces and mental models of the closest family and peers, can deceive seniors who only long for physical company. Furthermore, AI's capacity to perpetuate fraudulent activities underscores the sinister aspects of technological integration. In Singapore, videos of prominent figures such as former Prime Minister Lee have been manipulated for financial gain, illustrating how misinformation and private information can be compromised through clickbait (20). The proliferation of highly sophisticated deepfake technologies poses a significant threat, particularly to vulnerable groups who cannot distinguish mainstream media from fake outlets. This challenge is compounded by the cognitive decline that accompanies aging, resulting in a greater effort required for seniors to discern and judge the validity of incoming information (21). Moreover, their lack of digital literacy exacerbates their susceptibility to falling prey to such fraudulent schemes, causing immense financial losses, psychological trauma and a poignant breakdown in trust (21). These factors are likely to contribute to increased stress and a decline in AIDT uptake.

It is crucial to remain alert to the potential pitfalls of AIDTs and AI-generated content as they may lead to the dehumanization of medical care. The authors, as healthcare gatekeepers, acknowledge that AIDT is not a replacement for the human touch. As the saying goes, a kind gesture (human spirit) can reach a wound that only compassion can heal. AIDT-driven solutions have played a vital role in enhancing access to health information and promoting continuous health management, as demonstrated by digital literacy programmes amongst seniors (22). However, incorporating human-in-the-loop clinical decision-making is vital as there are significant concerns that AI could perpetuate biases and neglect the human elements. The AI Ethics Guidelines Global Inventory discusses how the lack of fair representation of seniors exacerbates the digital divide and leads to avoidable disparities. This occurs through the homogeneous grouping and reinforcement of negative stereotypes about cognitive and psychological decline in seniors (23). These concerns have become particularly pertinent when applying AIDTs to improve the quality of life for seniors through tools such as virtual games and digital inclusion programmes. Although these tools have been shown to improve cognition, extensively relying on them for emotional and social support could diminish meaningful human interactions. Such programmes should be complemented by existing social networks and opportunities that enhance social inclusion by augmenting the authenticity of physical connections. Turkle warns that electronic companions may reduce the quality of relationships and instead increase feelings of isolation and loneliness (24). Thus, despite the substantial benefits AIDTs offer, healthcare practitioners must ensure that these tools complement, rather than replace, the compassionate human touch essential for holistic human care.

Despite numerous digital inclusion programmes, there persists a misconception that seniors are technophobic and disinterested in adopting new technologies. In reality, however, they have shown eagerness and a strong desire to embrace the digital age (6). It is essential to critically examine whether current technological solutions effectively accommodate the diverse cognitive, sensory and physical needs of seniors, rather than perpetuating digital ageism through flawed design and implementation processes.

Unfortunately, biases are introduced in the training of generative AIDT models, including large language models, due to the lack of comprehensive and representative data on seniors. This stems from ineffective and incomplete data handling, as well as ageist assumptions that oversimplify older adults as a uniform group, without considering their immense diversity and nuanced lived experiences (6). In addition, sampling bias resulting from media under-representation and polarized age coding, which favors younger-associated adjectives, leads to semantic bias influenced by marketing and political sectors, skewing the usability and effectiveness of AIDT models (6). Shifting from a universal design approach to a participatory design, where seniors are empowered to advocate for their needs through active contribution to product development, can result in higher-quality products that are more readily accepted, increasing personalisation and lowering barriers to adoption.
